# Effectiveness of an interprofessional assessment and management approach for people with chronic low back disorders delivered via virtual care: A randomized controlled trial pilot intervention

**DOI:** 10.1177/20552076241260569

**Published:** 2024-06-05

**Authors:** Stacey Lovo, Biaka Imeah, Nazmi Sari, Megan E O’Connell, Steve Milosavljevic, Adriana Angarita-Fonseca, Brenna Bath

**Affiliations:** 1 School of Rehabilitation Science, 7235 University of Saskatchewan, Saskatoon, Saskatchewan, Canada; 2 Ministry of Social Services, 113243 Government of Saskatchewan, Regina, Saskatchewan, Canada; 3 Department of Economics, College of Arts and Science, 7235 University of Saskatchewan, Saskatoon, Saskatchewan, Canada; 4 College of Arts and Science, 7235 University of Saskatchewan, Saskatoon, Saskatchewan, Canada; 5 7001 Université du Québec en Abitibi-Témiscamingue, Rouyn Noranda, Quebec, Canada; 6 Centre de recherche du Centre hospitalier de l’Université de Montréal, 27952 Universidad de Santander, Bucaramanga, Colombia; 7 Canadian Center for Rural and Agricultural Health, 7235 University of Saskatchewan, Saskatoon, Canada

**Keywords:** Low back pain, telerehabilitation, rural health, physical therapy modalities, patient care team

## Abstract

**Objective:**

Virtual care for chronic conditions has seen uptake due to COVID-19. Evaluation of virtual models is important to ensure evidence-based practice. There is a paucity of research in the use of virtual care for management of chronic back disorders. The objective of this study was to evaluate effectiveness of a team-based virtual care model for back disorder assessment where a physical therapist uses virtual care to join a nurse practitioner and patient in a rural Saskatchewan, Canada community.

**Methods:**

Sixty-four rural adults with chronic back disorders were randomly allocated to receive either: (1) team-based virtual care (*n* = 24); (2) care from an urban physical therapist travelling to community (*n* = 20); or (3) care from a rural nurse practitioner (*n* = 20). The team-based care group involved a nurse practitioner located with a rural patient, and a physical therapist joining using virtual care. The physical therapist alone and the nurse practitioner alone groups received in-person assessments. Groups with a physical therapist involved had follow-up treatments by in-person physical therapy. Outcomes over six months included pain, disability, back beliefs, satisfaction, quality-adjusted health status and management-related costs.

**Results:**

There were no significant differences for pain, disability, back beliefs and satisfaction between groups. The average cost per patient for implementing in-person physical therapist assessment ($135) was higher compared with the team over virtual care ($118) and NP care ($59).

**Conclusion:**

Primary outcomes were not different by group. Physical therapist alone was more costly than other groups. Future research should include more participants, longer follow-up time and refined cost parameters.

**Trial Registration:**

ClinicalTrials.gov NCT02225535; https://clinicaltrials.gov/ct2/show/NCT02225535 (Archived by WebCite at http://www.webcitation.org/6lqLTCNF7).

## Introduction

Chronic low back disorders (CBD) are the most prevalent and costly musculoskeletal disorders in industrialized countries^1^ as well as the leading cause of disability worldwide.^2^ In Canada, the prevalence of CBD is nearly 20%.^3^ Rural and remote dwellers are 30% more likely to experience CBD^3^ and experience limited availability of specialized health care personnel and access to appropriate services. For example, 34% of Saskatchewan residents live in rural areas (outside of census metropolitan areas),^4^ while only 10% of physiotherapists work there.^5^ Patients in rural communities travel long distances and pay out-of-pocket travel costs in order to access physical therapists (PTs) and other specialist care in urban cities. Saskatchewan has a vast rural landscape, with variable geography and extremes of weather in summer (dry and hot) and winter (very cold with heavy snowfall and icy road conditions). This complexity of limited availability of health providers, long geographical distances from appropriate care, as well as travel cost barriers means reduced access to CBD care for rural and remote patients in Saskatchewan. New models of care to enhance access to services for CBD in rural and remote regions are needed.

Telehealth may provide a new approach to providing access to more appropriate care for CBD in rural and remote regions of Canada.^6^ Evidence is emerging for the use of virtual health for the PT management of musculoskeletal conditions.^7,8^ Lovo et al. reported a case study in which a PT using remote presence robotics joined a remote NP and her patient to complete a team-based neuromuscular assessment and a follow-up treatment for CBD. Outcomes included recovery of lumbar spine range of motion and lower extremity neural mobility.^9^ Further research examined the concordance of this team-based model of virtual care compared to in-person PT and in-person NP services alone. The diagnoses and management recommendations made by the team using telehealth were similar to those made by the in-person PT.^10^ Lovo et al. further described the experience of participants with a teams and technology model of care, finding 93.7% were very satisfied or satisfied with the virtual model of care, and reported patient and practitioner experiences included a theme of access to care supported by enhanced care for chronic back disorders, technology and interprofessional practice.^11^

To the best of our knowledge, there are no randomized controlled trials (RCTs) or economic evaluation studies evaluating the use of a team-based model of telehealth in the management of CBD. The objective of this study is to explore the difference between: (1) interprofessional team-based CBD assessment over videoconferencing (PT/NP_team_), (2) in-person PT assessment (PT_alone_) and (3) usual care delivered by a rural nurse practitioner (NP_alone_) (including PT in-person follow-up for the PT/NP_team_ and PT_alone_ groups) in the relevant outcomes and associated costs. We hypothesized that patients who had a team-based assessment for CBD where a PT joined by virtual means would have similar outcomes in pain, quality of life and perceived disability as care provided by a travelling PT, and improved outcomes compared to a usual care group.

## Methods

### Design and settings

This study was a pilot study. The design was a parallel RCT with three groups: (1) PT/NP_team_ where the PT joined the NP and patient using telehealth, (2) PT_alone_ and (3) NP_alone_. The study protocol has been previously published.^6^ The study took place in the communities of Arborfield and Carrot River, Saskatchewan which are 24.3 km apart and serviced by three NPs – two in Carrot River and one in Arborfield. This study was approved by the University of Saskatchewan Biomedical Ethics Board (#13-241).

### Participants

Patients between the ages of 18 and 80 years, and with back pain present for more than three months were invited to participate in the study. Patients were excluded if they were receiving third party payer funding (i.e. Worker's Compensation Board, or other) for their back-related complaints due to the fact that treatment requirements may be specified for third party payers which would complicate the nature of the research; had primarily neck (cervical spine) or mid back (thoracic spine) complaints; and had language, reading or comprehension barriers that would limit adequate completion of the study paperwork.^6^

### Recruitment and randomization

Participants were invited to participate using advertisements in local newspapers, social media (Facebook, Twitter), provision of study details through rural providers and posters at healthcare facilities and community centres. Seventy-six individuals that contacted us for the study gave their oral and written consent and agreement to participate. Among them, 12 people were excluded from the study due to exclusion criteria and the remaining 64 were randomly assigned to one of the three groups using simple block randomization. Randomization and assignment to groups was completed by a research assistant and was concealed through the use of envelopes. Researchers did not know about the assignment until the implementation began.

**Figure 1.**
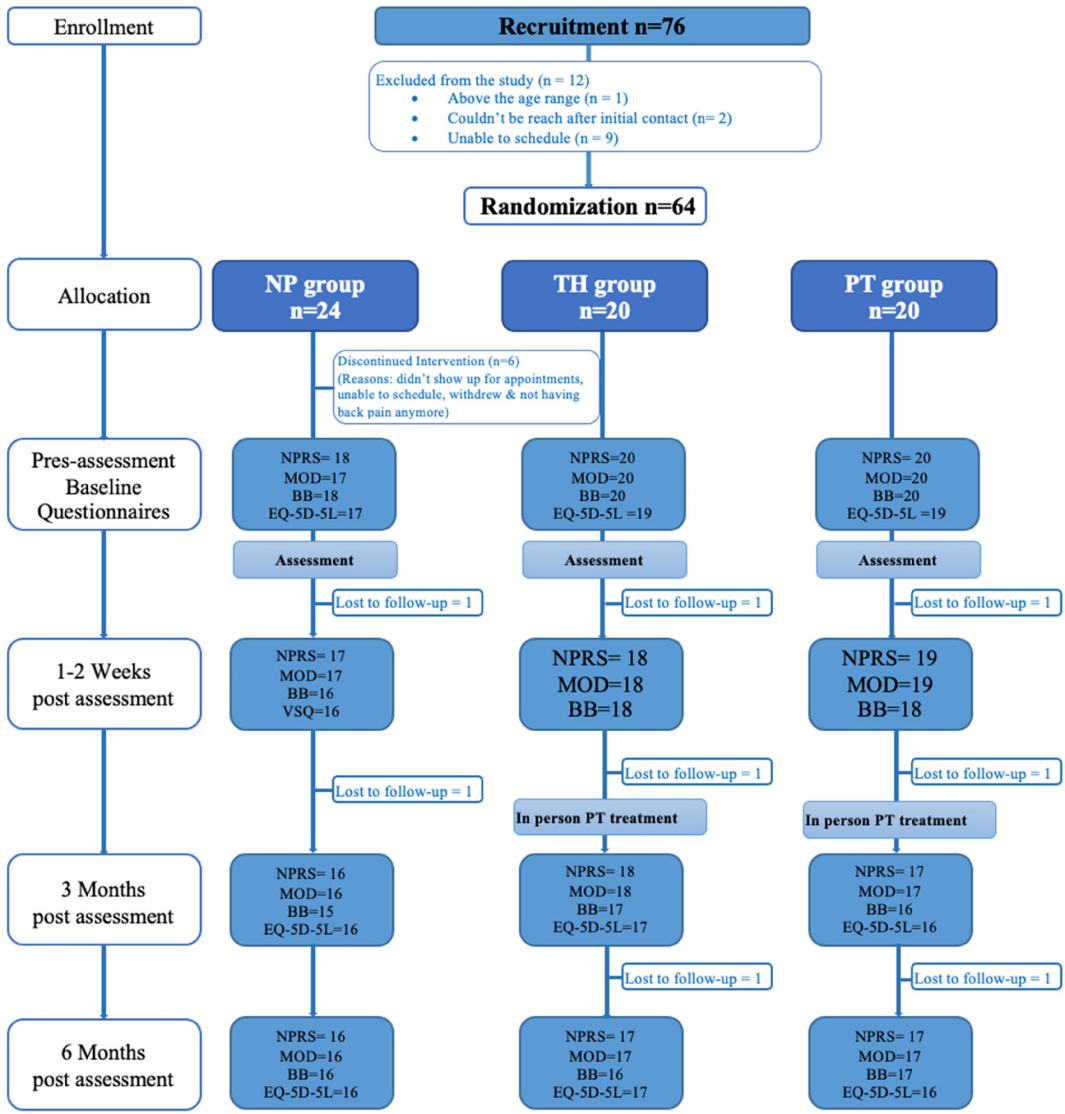
Flow diagram of participants’ progress during the randomized controlled trial period.

Six people discontinued from the study after being randomly assigned to the NP_alone_ group (reasons included not showing for appointment, inability to schedule, and withdrew and not having back pain anymore). This left a total of 58 participants at the baseline. Thus, the overall baseline participation rate among those who were eligible was 90.6% (58/64). At the end of the study we lost eight participants (moved, personal reasons, unable to contact them), and there was incomplete data in some questionnaires at each time-point. The study occurred between September 2016 and December 2019. Figure 1 depicts the flow of participants through each stage of the randomized trial.

NP_alone_: nurse practitioner; PT/NP_team_: telehealth; PT_alone_: physiotherapy; NPRS: Numeric Pain Rating Scale; MODI: Modified Oswestry Disability Index; BB: Back Beliefs Questionnaire; EQ-5D-5L: EuroQol health survey instrument; VSQ: Visit specific Satisfaction Questionnaire.

### Data collection

Data were collected in the rural communities using paper-based questionnaires over a six-month period including four time points: prior to receiving any form of health care services, within one to two weeks of the initial health care encounter, three months, and six months post baseline. All data was deidentified with a patient ID number so names and groups were not present on the completed questionnaires. Research assistants entered the data into statistical software using de-identified ID only, and statistical analysis was done using ID number only. Researchers were aware which intervention arm each ID was assigned to out of necessity for analysis. After the informed consent and baseline questionnaires, participants were assessed by healthcare professionals depending on the assigned group. If recommended on assessment, participants in the PT/NP_team_ and PT_alone_ groups received up to four follow-up sessions of in-person PT treatment after assessment.

### Groups

The usual care control group (NP_alone_) received care delivered by a rural NP to patients with CBD in the communities of Arborfield and Carrot River. NPs are primary healthcare providers in rural Saskatchewan, remunerated by the Health Authority, and manage caseloads independent of a physician oversight. NPs prescribe medications, order bloodwork, as well as order and interpret screening and diagnostic tests related to back pain.^12^

In-person PT assessment (PT_alone_) involved a PT who travelled 2.5 h each way to and from Arborfield to provide face-to-face care. Collaborative team-based health care services were delivered to patients with back pain via telehealth videoconferencing technologies (PT/NP_team_). The NP was located with the participant in person, and a PT joined using videoconferencing using a laptop with VidyoDesktop Software Inc.^13^ An external web camera with pan, tilt and zoom functionalities was located at the NP and patient site; this device transmitted audio and video to the consultant urban PT. The PT/NP_team_ neuromusculoskeletal assessment for the lumbar spine was completed in a collaborative and interprofessional manner (subjective history, physical examination, diagnosis, education and management recommendations). Both NPs and the PT involved in the team assessment model had more than 20 years of experience in their respective professions. The PT travelling to the community for face-to-face assessments and provision of follow-up PT care had over 10 years of experience and had a similar training history and practice setting as the PT from the PT/NP_team_. The PT intervention also involved subjective history, physical examination, diagnosis, education and management recommendations.

### Variables

Baseline data included demographics (age, gender, marital status, body mass index); distance travelled; socio-economic variables, (family income, work status) general health characteristics (smoking status, number of comorbidities, location of the pain); and psychological factors (i.e. fear avoidance beliefs,^14^ and distress and risk assessment which combines depression and somatization scores).^14^^,^^15^ Outcome measures evaluated at four time points (i.e. baseline, one–two weeks, three months and six months post initial assessment) were: perceived disability;^16^^,^^17^ pain;^18^ and back beliefs.^19^ Detailed data on costs of healthcare services (direct and indirect), and visit specific satisfaction of participants^19^ were also collected.

### Outcome measures

The *Modified Oswestry Disability Index (MODI)* questionnaire consists of ten categories addressing: pain intensity, personal care, lifting, walking, sitting, standing, sleeping, sex life, social life, and travelling. Each of the categories has six statements (that describe the problem associated with that category) from which patients are requested to select one. The statements are scored on a scale of 0–5, with 5 representing the greatest disability. This allows a maximum possible score of 50. The total score of a patient was then divided by the maximum possible score (50) and multiplied by 100 to create a percentage disability score.^17^

Using the MODI score, we examined the proportion of participants achieving clinically meaningful improvement. Ostelo et al. (2008) recommended a change of 10 in the MODI score (from baseline to follow-up period) as a threshold for identifying clinically meaningful improvement.^20^ Participants in the control and intervention groups were divided into two groups (based on change in their MODI score between periods): MODI score change <10 and MODI score change ≥10).

The *Numeric Pain Rating Scale (NPRS)* is an 11-point numeric scale ranging from ‘0’ representing no pain to ‘10’ representing pain as bad as it could be. The score was obtained by averaging the answers about current, worst and least pain in the past 24 h.^18^

The *Back Beliefs Questionnaire (BB) c*onsists of 14 questions. Nine items are used for the score, and five are used as distractors. It uses a Likert 5-level scale ranging from completely disagree to completely agree. The score was obtained by adding the responses to the 9 items. A higher score means a lower propensity for fear and false beliefs.^19^

The *EQ-5D-5L* consists of a descriptive system and a visual analogue scale.^21^ The descriptive system captures five dimensions of health-related quality of life: mobility, self-care, usual activities, pain/discomfort, sand anxiety/depression. Each dimension has five levels labelled: no problems, slight problems, moderate problems, severe problems and extreme problems. To score the descriptive system, ‘the respondent is asked to indicate his or her health states by checking the box against the most appropriate statement in each of the five dimensions’.^21^ The visual analogue on page two of the EQ-5D-5L records the respondent self-rated health on a scale of zero to one hundred with zero as the worst health state and one hundred as the perfect health state.

The *Visit-Specific Satisfaction* Instrument (VSQ) consisted of nine questions and used a 5-point Likert scale of excellent, very good, good, fair and poor. To score the VSQ, the responses from each individual were transformed linearly to a 0 to 100 scale, with 100 corresponding to ‘excellent’, 75 to ‘very good’, 50 to ‘good’, 25 to ‘fair’ and 0 to ‘poor’. Responses to the nine VSQ items were then averaged together to create a VSQ-9 ‘overall score’ for each person.^22^

### Statistical analysis

Data were coded to permit blinding of researchers to group allocation during statistical analysis. Descriptive statistics were computed for selected demographic and clinical characteristics. A Shapiro Francia Test was used to determine the normal distribution of all continuous variables. Non-normal distributed variables were presented as median and interquartile range.

Means and 95% confidence intervals (95% CIs) were reported for continuous variables by group and time point (pain, disability and beliefs), while categorical variables were presented as raw counts (number, percentage). Changes from baseline at each time point were also reported as means with 95% CIs and illustrated in graphs. Effect size (Cohen’s *d*) calculation and 95% CI were calculated for the mean differences between groups. Effect sizes of 0.2, 0.5 and 0.8 were considered to correspond to small, medium and large differences respectively.^23^

## Results

### Baseline characteristics

Table 1 shows the participant characteristics at baseline by group. The three trial arms were well-balanced except for work status (Table 1). The proportion of people with paid work-full time and paid work-part time was higher in PT/NP_team_ and PT_alone_ groups respectively. Unintended harms and effects due to assessment or treatment were not identified for any group.

**Table 1.** Characteristics of study participants at baseline by intervention group.

Variable		Group					
		NP_alone_		PT/NP_team_		PT_alone_	
Demographics	Age *Mean SD*	60.8	11.4	51.4	13.7	53.6	12.5
	Gender						
	Female	11	61.1	11	55.0	12	60.0
	Male	7	38.9	9	45.0	8	40.0
	Education						
	No grade 12	5	29.4	7	35.0	6	30.0
	High school	5	29.4	4	20.0	5	25.0
	Trade school	3	17.7	4	20.0	6	30.0
	Some University/University Degree/Graduate Degree	4	23.5	5	25.0	3	15.0
	Income						
	<30,000	4	25.0	5	26.3	5	27.8
	30000–60000	9	56.3	10	52.6	8	44.4
	>60000	3	18.7	4	21.1	5	27.8
	Marital status						
	Married	15	83.3	15	75.0	14	73.7
	Divorced/Widowed/Never Married	3	16.7	5	25.0	5	26.3
	Work status						
	Paid work-full time	4	23.5	9	45.0	7	35.0
	Paid work-part time	2	11.8	5	25.0	10	50.0
	Housework	5	29.4	2	10.0	2	10.0
	Retired	6	35.3	4	20.0	1	5.0
	Distance travelled for assessment (km)						
	<15	10	62.5	9	50.0	6	31.6
	15–30	4	25.0	4	22.2	9	47.4
	>30	2	12.5	5	27.8	4	21.0
General health	BMI classification	n	%	n	%	n	%
	Normal	3	16.7	4	20.0	5	25.0
	Overweigh	8	44.4	7	35.0	6	30.0
	Obesity	7	38.9	9	45.0	9	45.0
	Smoking						
	Never	9	50.0	9	45.0	8	40.0
	Used to	6	33.3	8	40.0	10	50.0
	Smoker	3	16.7	3	15.0	2	10.0
	Number of comorbidities						
	0	1	5.5	3	15.0	3	15.0
	1–2	14	77.8	13	65.0	13	65.0
	3–4	3	16.7	4	20.0	4	20.0
Psychological variables	DRAM						
	Normal	10	55.6	12	60.0	11	55.0
	At risk	6	33.3	4	20.0	2	10.0
	Distressed	2	11.1	4	20.0	7	35.0
	FABQP *Mean SD*	10.1^a^	5.9	12.1^b^	6.0	10.1^b^	5.6
	FABQW *Mean SD*	13.6^c^	9.7	18.1^c^	7.6	12.7^d^	9.5
Back Health							
	Back pain only						
	No	12	70.6	14	70.0	18	90.0
	Yes	5	29.4	6	30.0	2	10.0
	Pain above knee						
	No	13	76.5	13	65.0	9	45.0
	Yes	4	23.5	7	35.0	11	55.0
	Pain in another site						
	No	10	58.8	10	50.0	7	35.0
	Yes	7	41.2	10	50.0	13	65.0
	Symptom total duration (years)						
	≤1	2	11.8	2	11.1	2	10.0
	1.1–10	9	52.9	7	38.9	7	35.0
	10.1–55	6	35.3	9	50.0	11	55.0

BMI: body mass index; DRAM: distress and risk assessment method.

^a^*n* = 18.

^b^*n* = 19.

^c^*n* = 14.

^d^*n* = 17.

### Pain, disability and back beliefs

**Figure 2.**
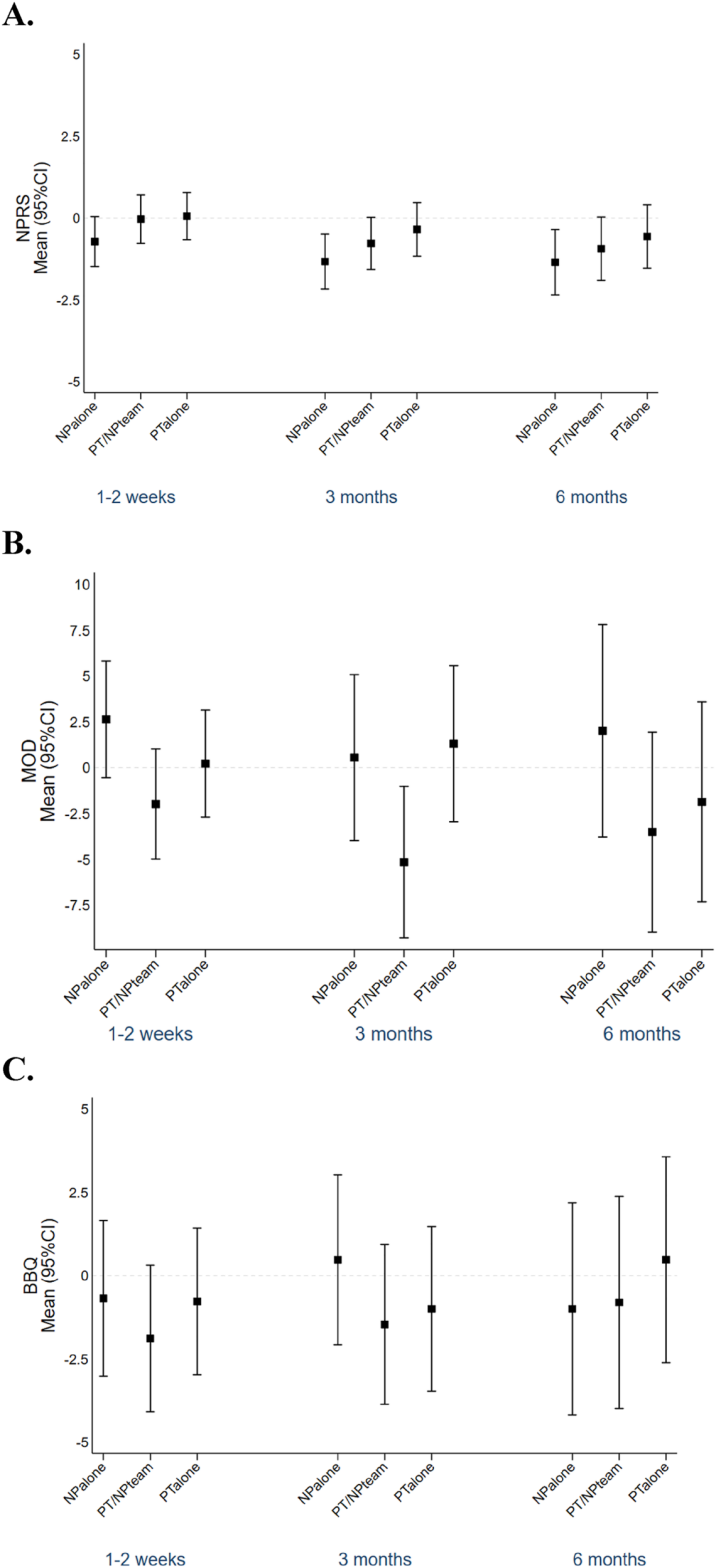
Mean of differences between baseline and 1–2 weeks, 3 months and 6 months for each group. A. Pain. B. Disability. C. Back beliefs.

Within-group changes for pain, disability and back beliefs from baseline over time generally showed no significant changes, except for reductions in pain at three and six months in the NP alone group, and a significant reduction in disability at three months in the PT/NP team group (Table 2). However, when comparing between groups – NP_alone_, PT/NP_team_ and PT_alone_ – there were no statistically significant differences in outcomes for pain, disability or back beliefs at any assessed time points (Table 2). Mean differences at each time point compared to baseline were not significantly different between groups for pain, disability and back beliefs (Figure 2). Most of the effect sizes were between small and medium with a Cohen's *d* less than 0.50 (Table 3).

**Table 2.** Pain, disability and back belief at baseline, 1–2 weeks, 3 and 6 months post assessment by intervention group.

Variable	Group					
	NP_alone_		PT/NP_team_		PT_alone_	
	*Mean*	*95% CI*	*Mean*	*95% CI*	*Mean*	*95% CI*
**NPRS (pain)**	** **	** **	** **	** **	** **	** **
Baseline	4.53	3.71, 5.37	4.52	3.71, 5.34	4.40	3.58, 5.22
1–2 weeks	3.69	2.83, 4.54	4.50	3.51, 5.49	4.33	3.44, 5.22
3 months	3.02	2.30, 3.74	3.69	2.88, 4.49	4.04	3.06, 5.02
6 months	3.00	2.05, 3.95	3.47	2.74, 4.20	3.90	2.61, 5.19
Δ 1–2 weeks versus baseline	−0.73	−1.74, 0.29	−0.04	−0.64, 0.56	0.05	−0.70, 0.81
Δ 3 months versus baseline	−1.33	−2.40, −0.26	−0.78	−1.62, 0.07	−0.35	−1.08, 0.38
Δ 6 months versus baseline	−1.35	−2.32, −0.39	−0.94	−2.01, 0.13	−0.57	−1.69, 0.55
**I (disability)**						
Baseline	20.59	14.49, 26.69	25.11	20.24, 29,97	23.30	18.44, 28.15
1–2 weeks	23.05	17.24, 28.88	23.33	17.23, 29,44	23.26	17.89, 28.63
3 months	20.38	14.47, 26,28	19.38	13.92, 24,84	24.47	16.84, 32.10
6 months	21.38	15.30, 27.45	20.12	13.50, 26.74	21.76	12.50, 31.03
Δ 1–2 weeks versus baseline	2.63	−1.98, 6.33	−2.00	−4.53, 0.53	0.21	−3.29, 3.71
Δ 3 months versus baseline	0.53	−3.40, 4.47	−5.17	−10.33, −0.02	1.29	−3.22, 5.80
Δ 6 months versus baseline	2.00	−1.70, 5.70	−3.5	−9.93, 2.87	−1.88	−8.87, 5.11
**BB (back beliefs)**						
Baseline	29.44	27.0, 31.88	29.15	26.16, 32.14	30.10	27.59, 32.61
1–2 weeks	29.19	26.42, 31.95	27.50	23.99, 31.01	30.06	27.55, 32.57
3 months	29.53	26.40, 32.67	27.88	24.22, 31.54	29.56	26.82, 32.31
6 months	28.19	23.94, 32.43	28.75	25.81, 32.83	30.82	28.81, 32.83
Δ 1–2 weeks versus baseline	−0.69	−3.18, 1.81	−1.89	−4.62, 0.84	−0.78	−2.76, 1.20
Δ 3 months versus baseline	0.47	−1.87, 2.81	−1.47	−3.73, 0.79	−1.00	−4.32, 2.32
Δ 6 months versus baseline	−1.00	−5.06, 3.06	−0.81	−3.38, 1.76	0.47	−2.97, 3.91

**Table 3.** Cohen's *d* effect size of assessment groups on pain, disability and back beliefs.

Variable	Group					
	NP_alone_ versus PT/NP_team_		PT/NP_team_ versus PT_alone_		NP versus PT_alone_	
	***MD *± SD**	***d 95% CI***	***MD *± SD**	***d 95% CI***	***MD *± SD**	***d 95% CI***
**NPRS**	** **	** **	** **	** **	** **	** **
Baseline	0.01 ±	0.01	0.13 ±	0.07	0.14 ±	0.08
	0.56	−0.63; 0.64	0.55	−0.55; 0.69	0.56	−0.56; 0.72
1–2 weeks	−0.814 ±	−0.44	0.17±	0.09	−0.65 ±	−0.37
	0.622	−1.11; 0.23	0.63	−0.56; 0.73	0.59	−1.02; 0.29
3 months	−0.66 ±	−0.44	−0.35 ±	−0.20	−1.02 ±	−0.61
	0.52	−1.12; 0.24	0.60	−0.86; 0.46	0.58	−1.31; 0.09
6 months	−0.47 ±	−0.29	−0.43 ±	−0.21	−0.90 ±	−0.41
	0.56	−0.98; 0.39	0.70	−0.88; 0.46	0.76	−1.09; 0.28
**MOD**						
Baseline	−4.52±	−0.41	1.81±	0.17	−2.71±	−0.25
	3.66	−1.06; 0.25	3.28	−0.45; 0.79	3.65	-.89; 0.41
1–2 weeks	−0.274±	−0.02	0.07±	0.006	−0.20±	−0.02
	4.00	−0.69; 0.64	3.84	−0.64; 0.65	3.75	−0.67; 0.64
3 months	0.99±	0.09	−5.09 ±	0.006	−4.09±	−0.311
	3.79	−0.58; 0.76	4.39	−0.638; 0.650	4.58	−0.99; 0.37
6 months	1.25 ±	0.10	−1.65±	−0.11	−0.39±	-.026
	4.24	-.58; 0.79	5.37	−0.78; 0.57	5.29	−0.71; 0.66
**BB**						
Baseline	0.29±	0.05	−0.95 ±	−0.16	−0.66 ±	−0.13
	1.86	−0.59; 0.69	1.86	−0.78; 0.46	1.67	−0.76; 0.51
1–2 weeks	1.69±	0.27	−2.56 ±	−0.42	−0.87±	−0.17
	2.15	−0.41; 0.9	2.05	−1.07; 0.25	1.76	−0.84; 0.51
3 months	1.65 ±	0.25	−1.68 ±	−0.27	−0.03±	−0.005
	2.29	−0.45; 0.95	2.18	−0.95; 0.42	1.94	−0.71; 0.70
6 months	−0.56±	−0.078	−2.07 ±	−0.39	−2.6±	−0.42
	2.55	−0.77; 0.62	1.83	−1.08; 0.30	2.16	−1.11; 0.27

MD: mean of differences; SD: standard deviation; d: Cohen's *d* effect size.

### Implementation costs

The intervention has three cost components: the cost of implementing usual care, telehealth and in-person PT treatment. The cost of implementing usual care comprises the hourly wages of the NP and receptionist. The cost of implementing telehealth (PT/NP_team_) included: hourly wages of PT and NP, the cost of the telehealth system, and the cost of PT and NP standardization training. The costs of implementing in-person PT assessment include: hourly wage of PT and travel costs.

The average cost per patient for implementing in-person PT assessment ($135) compared with a PT/NP_team_ over telehealth ($118) or care from a NP ($59) was higher. PT travel cost and time was an important cost driver for the PT_alone_ intervention.

### Clinically meaningful improvement on MODI

From baseline to six months follow-up, 13.3% of the NP_alone_, 11.8% of the PT/NP_team_ and 17.7% of the PT_alone_ group achieved clinically meaningful improvement (Table 4).

**Table 4.** Clinically meaningful improvement on MODI at 6 months.

Group	Clinically meaningful improvement (MODI≥10)			
	No		Yes	
	*n*	%	*n*	%
NP_alone_	13	86.7	2	13.3
PT/NP_team_	15	88.2	2	11.8
PT_alone_	14	82.3	3	17.7

### Sample size calculation for future studies

This study was a pilot study, thus an a priori sample size was not determined. The estimated sample size for repeated-measures ANOVA was calculated using an F-test for between-within subjects with Greenhouse-Geisser correction, alpha of 0.05, power of 0.80, delta of 0.39, two-tailed test, three groups, four measurements, and means and covariances. The approximate sample size needed for future studies using the same design and intervention to achieve adequate power is 111, with 37 participants for each group.

## Discussion

To our knowledge, this study represents the first research investigating a team-based assessment in people with CBD using telerehabilitation. This study examined the effect and implementation costs of an interprofessional team assessment using telehealth (PT/NP_team_), and in-person PT assessment compared to usual care from a NP in people with CBD in a rural Saskatchewan community. The groups who had PT as part of their assessment also had follow-up PT. The effects of being assessed by PT/NP_team_, PT_alone_ (and follow up PT treatment for these groups) or NP_alone_ on pain, disability, back belief and perceived disability were not statistically different by group. The implementation cost per patient for the NP group ($59) was smaller than the TH group ($118) and the PT group ($135). PT travel cost and time was an important cost driver for the PT groups.

One limitation of the study is the issue of small sample size. As this is a pilot study the typical sample size is small. Due to small sample size and lack of statistical power, one cannot infer that the intervention doesn’t offer any benefit.

Second, three months and six months follow-up period provided a short-term perspective of the effects of treatments on CBD. The duration is not significantly long enough to capture all of the clinical ramifications that could affect the benefits associated with the interventions, especially in the case of CBD which can last over months and years of a person's lifetime. Benefits of the interventions are likely to have been underestimated due to the short-term follow-up period. Future studies should extend the trial period to more than six months.

The study was unblinded to participants since they knew which assessment group they were in. Theoretically, this could lead to a reporting bias depending on the belief and previous experiences with NP or PT health providers. A negative healthcare experience in the past could affect the outcome evaluation in this study.

The gender and age distribution in this study is a notable limitation. The majority of participants were women and most were above 50 years. We could not, therefore, generalize to males or people under the age of 50. As well, due to the long wait list in the communities for back pain care, the majority of participants had been dealing with CBD for a large number of years and may not have been actively seeking care for their chronic condition when this project occurred. Results may differ with patients actively seeking care for CBD.

One final limitation is that although costs were measured for each of the groups, cost-effectiveness was not assessed and this will be an important component for future study, to ensure the ability to inform policy and practice.

### Generalizability of the results

This study was developed in a small convenience sample from a rural community of Saskatchewan, Canada. Because health service provision across the different rural settings depends on the availability of economic and human resources, the results cannot be generalized to the broader rural Canadian population or other countries with different health systems and technological availability.

## Conclusion

We did not identify differences in effect of intervention by group for pain, perceived disability or back beliefs. The implementation cost of telehealth (PT/NP_team_) was higher than that of usual NP care. PT_alone_ was most costly due to the travel incurred from an urban centre to a rural community 2.5 h away. This study provides information for future research of virtual care interventions for the management of CBD by healthcare teams.

Due to the limitations of the study design, further research should include larger participant numbers in a RCT in rural communities with CBD, and refinement of cost variables.
